# Supplementary material for: Doxorubicin-loaded human serum albumin nanoparticles overcome transporter-mediated drug resistance in drug-adapted cancer cells
Source: Beilstein J Nanotechnol. 2019 Aug 14;10:1707–15. doi: 10.3762/bjnano.10.166 (PMC6720578; doi:10.3762/bjnano.10.166)
Supplement: File 1 — Additional experimental details. [file Beilstein_J_Nanotechnol-10-1707-s001.pdf]

BEILSTEIN JOURNAL OF NANOTECHNOLOGY

# Supporting Information

for

# Doxorubicin-loaded human serum albumin nanoparticles overcome transporter-mediated drug resistance in drug-adapted cancer cells

Hannah Onafuye, Sebastian Pieper, Dennis Mulac, Jindrich Cinatl Jr., Mark N. Wass, Klaus Langer and Martin Michaelis

*Beilstein J. Nanotechnol.* **2019,** *10,* 1707–1715. doi:10.3762/bjnano.10.166

# Additional experimental details

License and Terms: This is a supporting information file under the terms of the Creative Commons Attribution License (http://creativecommons.org/licenses/by/4.0). Please note that the reuse, redistribution and reproduction in particular requires that the authors and source are credited.
The license is subject to the *Beilstein Journal of Nanotechnology* terms and conditions: (https://www.beilstein-journals.org/bjnano)

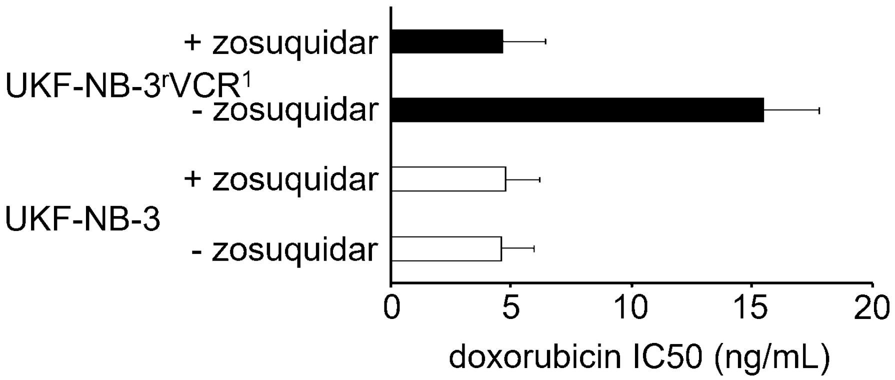


**Figure S1:** Doxorubicin concentrations that reduce neuroblastoma cell viability by 50% (IC50) in the absence or presence of the ABCB1 inhibitor zosuquidar (1 µM).

**Table S1:** Effects of doxorubicin (Dox) applied as solution or incorporated into human serum albumin (HSA) nanoparticles on neuroblastoma cell viability. The investigated nanoparticles differed in the amount of the crosslinker glutaraldehyde that was used for nanoparticle stabilisation. The glutaraldehyde amount corresponded to 40% (Dox HSA(40%) NP), 100% (Dox HSA(100%) NP), or 200% (Dox HDA(200%) NP) of the theoretical amount of available amino groups present on HSA. Preparations prepared without glutaraldehyde served as control (Dox HSA(0%) NP). Values are expressed as concentrations that reduce cell viability by 50% (IC50) as determined by MTT assay after 120 h of incubation.

| | IC50 doxorubicin (ng/mL) | | |
|---|---|---|---|
| | UKF-NB-3 | UKF-NB-3$^r$DOX$^{20}$ | UKF-NB-3$^r$VCR$^1$ |
| Dox solution | 3.85 ± 2.46 | 89.0 ± 30.8 (23.1)[a] | 15.5 ± 2.3 (4.03)[a] |
| DoxHSA(0%) | 4.20 ± 1.72 (1.09)[b] | >200[c] (>2.25)[b] | 9.88 ± 3.78 (0.64)[b] |
| DoxHSA(40%) | 1.55 ± 1.00 (0.40)[b] | 42.8 ± 13.3 (0.48)[b] | 4.25 ± 1.35 (0.27)[b] |
| DoxHSA(100%) | 1.98 ± 1.03 (0.51)[b] | 39.1 ± 18.6 (0.44)[b] | 3.52 ± 2.00 (0.23)[b] |
| DoxHSA(200%) | 1.78 ± 1.04 (0.46)[b] | 31.2 ± 12.9 (0.35)[b] | 3.51 ± 1.66 (0.23)[b] |

[a] fold change in doxorubicin sensitivity relative to UKF-NB-3
[b] fold change in doxorubicin sensitivity relative to doxorubicin solution
[c] cell viability in the presence of doxorubicin 200 ng/mL applied as non-stabilised HSA preparation: 81.9 ± 12.9% relative to untreated control

**Table S2:** Effects of doxorubicin (Dox) applied as solution or incorporated into human serum albumin (HSA) nanoparticles on neuroblastoma cell viability in the absence or presence of zosuquidar (1 µM). The investigated nanoparticles differed in the amount of the crosslinker glutaraldehyde that was used for nanoparticle stabilisation. The glutaraldehyde amount corresponded to 40% (Dox HSA(40%) NP), 100% (Dox HSA(100%) NP), or 200% (Dox HDA(200%) NP) of the theoretical amount of available amino groups present on HSA. Values are expressed as concentrations that reduce cell viability by 50% (IC50) as determined by MTT assay after 120 h of incubation.

| UKF-NB-3 | | + Zosuquidar (1 µM) | | |
|---|---|---|---|---|
| | Doxorubicin IC50 (ng/mL) | Zosuquidar alone[1] | Doxorubicin IC50 (ng/mL) | Fold change[2] |
| Doxorubicin | 4.80 ± 1.41 | 107 ± 24 | 4.64 ± 1.33 | 1.04 |
| Dox HSA (40%) NP | 2.01 ± 1.40 | 107 ± 24 | 2.52 ± 0.11 | 0.80 |
| DOX HSA (100%) NP | 2.61 ± 1.11 | 107 ± 24 | 3.48 ± 1.31 | 0.75 |
| DOX HSA (200%) NP | 2.34 ± 1.35 | 107 ± 24 | 3.70 ± 0.86 | 0.63 |

| UKF-NB-3$^r$DOX$^{20}$ | | + Zosuquidar (1 µM) | | |
|---|---|---|---|---|
| | Doxorubicin IC50 (ng/mL) | Zosuquidar alone[1] | Doxorubicin IC50 (ng/mL) | Fold change[2] |
| Doxorubicin | 91.0 ± 15.9 | 112 ± 17 | 36.9 ± 7.7 | 2.47 |
| Dox HSA (40%) NP | 30.5 ± 2.4 | 112 ± 17 | 17.4 ± 0.3 | 1.75 |
| DOX HSA (100%) NP | 29.3 ± 12.2 | 112 ± 17 | 19.3 ± 2.5 | 1.52 |
| DOX HSA (200%) NP | 20.1 ± 14.4 | 112 ± 17 | 17.7 ± 0.6 | 1.14 |

| UKF-NB-3$^r$VCR$^1$ | | + Zosuquidar (1 µM) | | |
|---|---|---|---|---|
| | Doxorubicin IC50 (ng/mL) | Zosuquidar alone[1] | Doxorubicin IC50 (ng/mL) | Fold change[2] |
| Doxorubicin | 15.5 ± 2.3 | 99 ± 13 | 4.69 ± 1.75 | 3.31 |
| Dox HSA (40%) NP | 4.25 ± 1.35 | 99 ± 13 | 5.21 ± 0.91 | 0.82 |
| DOX HSA (100%) NP | 3.52 ± 2.00 | 99 ± 13 | 3.92 ± 1.08 | 0.90 |
| DOX HSA (200%) NP | 3.51 ± 1.66 | 99 ± 13 | 4.01 ± 0.84 | 0.87 |

[1] cell viability in the presence of Zosuquidar (1 µM) expressed as % untreated control
[2] doxorubicin IC50/ Doxorubicin IC50 in the presence of zosuquidar
